# Supplementary material for: Chimpanzees organize their social relationships like humans
Source: Sci Rep. 2022 Oct 5;12:16641. doi: 10.1038/s41598-022-20672-z (PMC9534840; doi:10.1038/s41598-022-20672-z)
Supplement: Supplementary file 1 — Supplementary Information. [file 41598_2022_20672_MOESM1_ESM.pdf]

Chimpanzees organize their social relationships like humans

Diego Escribano,[1] Victoria Doldán-Martelli,[1] Katherine A. Cronin,[2,3] Daniel B.M. Haun,[3] Edwin J.C. van Leeuwen,[4] José A. Cuesta,[1,5] and Angel Sánchez[1,5]

[1]Grupo Interdisciplinar de Sistemas Complejos (GISC), Departamento de Matemáticas, Universidad Carlos III de Madrid, 28911 Leganés, Spain

[2]Animal Welfare Science Program, Lincoln Park Zoo,Chicago, IL 60614, USA

[3]Lester E. Fisher Center for the Study and Conservation of Apes, Lincoln Park Zoo, 60614 Chicago, IL, USA

[4]Department for Comparative Cultural Psychology, Max Planck Institute for Evolutionary Anthropology, Deutscher Platz 6, 04103 Leipzig, Germany

[5]5Leipzig Research Centre for Early Child Development, Leipzig University, Jahnallee 59, 04109 Leipzig, Germany

[6]Animal Behaviour and Cognition, Department of Biology, University Utrecht, 3584 CH Utrecht, The Netherlands

[7]Instituto de Biocomputación y Física de Sistemas Complejos (BIFI), Universidad de Zaragoza, 50018 Zaragoza, Spain

Supplementary information

Chimpanzees info

Group	Individual	Sex	Age*	Origin	Subspecies	Parameter η
1	Pal	Male	39	Wild born	Schweinfurthii	Yes
1	Booboo	Male	38	Wild born	Schweinfurthii	Yes
1	Tobar	Male	38	Wild born	Verus	Yes
1	Girly	Female	38	Wild born	Schweinfurthii	Yes
1	Rita	Female	37	Wild born	Schweinfurthii	Yes
1	Tara	Male	37	Wild born	Schweinfurthii	Yes
1	Ingrid	Female	29	Captive born	Schweinfurthii	Yes
1	Brenda	Female	24	Captive born	Schweinfurthii	Yes
1	Genny	Female	23	Captive born	Schweinfurthii	Yes
1	Renate	Female	23	Captive born	Schweinfurthii	Yes
1	Bob	Male	19	Captive born	Schweinfurthii	Yes
1	Gerard	Male	18	Captive born	Unknown	Yes
1	Ilse	Female	17	Captive born	Unknown	Yes
1	Chrissie	Female	13	Captive born	Unknown	Yes
1	Rusty	Male	13	Captive born	Unknown	Yes
1	Regina	Female	13	Captive born	Unknown	Yes
1	Innocentia	Female	13	Captive born	Unknown	Yes
1	BJ	Female	12	Captive born	Unknown	Yes
1	Gonzaga	Female	12	Captive born	Unknown	Yes
1	Irene	Female	8	Captive born	Unknown	Yes
1	Rachel	Female	7	Captive born	Unknown	Yes
1	Richard	Male	5	Captive born	Unknown	No
1	Ian	Male	5	Captive born	Unknown	No
1	Genny's baby	Female	4	Captive born	Unknown	No
1	Ida	Female	4	Captive born	Unknown	No
1	Ricky	Male	1	Captive born	Unknown	No

Table S1: Group 1 info. *Age at the end of the observation window (late 2019)

Group	Individual	Sex	Age*	Origin	Subspecies	Parameter η
2	Noel	Female	43	Wild born	Schweinfurthii	Yes
2	Donna	Female	36	Wild born	Schweinfurthii	Yes
2	Little Jane	Female	35	Wild born	Schweinfurthii	Yes
2	Coco	Female	35	Wild born	Schweinfurthii	Yes
2	Maggie	Female	34	Wild born	Schweinfurthii	Yes
2	Misha	Female	32	Wild born	Schweinfurthii	Yes
2	Mikey	Male	32	Wild born	Schweinfurthii	No
2	Pan	Male	31	Wild born	Schweinfurthii	Yes
2	Pippa	Female	31	Wild born	Schweinfurthii	Yes
2	Dora	Female	31	Wild born	Schweinfurthii	Yes
2	Trixie	Female	30	Wild born	Schweinfurthii	Yes
2	Zsabu	Male	30	Wild born	Schweinfurthii	Yes
2	Masya	Female	29	Wild born	Schweinfurthii	Yes
2	Violet	Female	29	Wild born	Schweinfurthii	No
2	Diana	Female	29	Wild born	Schweinfurthii	Yes
2	Little Judy	Female	24	Captive born	Unknown	Yes
2	Dolly	Female	23	Captive born	Schweinfurthii	Yes
2	Carol	Female	23	Captive born	Unknown	Yes
2	Nikkie	Female	22	Captive born	Schweinfurthii	Yes
2	Tess	Female	21	Captive born	Unknown	Yes
2	Tilly	Female	19	Captive born	Unknown	Yes
2	Debbie	Female	18	Captive born	Unknown	Yes
2	David	Male	18	Captive born	Unknown	Yes
2	Maxine	Female	18	Captive born	Unknown	No
2	Doug	Male	17	Captive born	Unknown	Yes
2	Nina	Female	17	Captive born	Unknown	Yes
2	Claire	Female	17	Captive born	Unknown	Yes
2	Toni	Female	17	Captive born	Unknown	No
2	Vis	Male	16	Captive born	Unknown	Yes
2	Taylor	Female	16	Captive born	Unknown	No
2	Daisey	Female	15	Captive born	Unknown	Yes
2	Mary	Female	14	Captive born	Unknown	No
2	Long John	Male	13	Captive born	Unknown	Yes
2	Little Jenkins	Female	13	Captive born	Unknown	Yes
2	Max	Male	13	Captive born	Unknown	Yes
2	Darwin	Male	13	Captive born	Unknown	No
2	Dizzy	Female	12	Captive born	Unknown	Yes
2	Moyo	Male	12	Captive born	Unknown	Yes
2	Charity	Female	12	Captive born	Unknown	Yes
2	Little Jones	Female	9	Captive born	Unknown	No
2	Little Jacky	Male	8	Captive born	Unknown	No
2	Danny	Male	8	Captive born	Unknown	No
2	Martin	Male	8	Captive born	Unknown	No
2	May	Female	7	Captive born	Unknown	No
2	Masya's baby	Female	7	Captive born	Unknown	No
2	Mavis	Female	7	Captive born	Unknown	No
2	Chitalu	Female	6	Captive born	Unknown	No
2	Debbie's baby	Male	5	Captive born	Unknown	No
2	Tom	Male	5	Captive born	Unknown	No
2	Tina	Female	4	Captive born	Unknown	No
2	Don	Male	4	Captive born	Unknown	No
2	Toni's baby	Female	4	Captive born	Unknown	No
2	Nina's baby2	Female	4	Captive born	Unknown	No
2	Mumba	Male	3	Captive born	Unknown	No
2	Nancy	Female	3	Captive born	Unknown	No
2	Merial	Female	2	Captive born	Unknown	No
2	Little Joey	Male	2	Captive born	Unknown	No
2	Camilla	Female	2	Captive born	Unknown	No
2	Muriel	Female	2	Captive born	Unknown	No
2	Victoria	Female	2	Captive born	Unknown	No

Table S2: Group 2 info. *Age at the end of the observation window (late 2019)

Group	Individual	Sex	Age*	Origin	Subspecies	Parameter η
3	Buffy	Female	35	Wild born	Schweinfurthii	Yes
3	Clement	Male	27	Wild born	Schweinfurthii	Yes
3	Brian	Male	26	Wild born	Schweinfurthii	Yes
3	E.T.	Female	25	Wild born	Schweinfurthii	Yes
3	Roxy	Female	25	Wild born	Schweinfurthii	Yes
3	Barbie	Female	24	Wild born	Schweinfurthii	Yes
3	Bussie	Male	15	Captive born	Unknown	Yes
3	Bruce	Male	10	Captive born	Unknown	Yes
3	Lods	Female	9	Captive born	Unknown	Yes
3	Brent	Female	6	Captive born	Unknown	Yes
3	Bill	Male	1	Captive born	Unknown	No

Table S3: Group 3 info. *Age at the end of the observation window (late 2019)

Group	Individual	Sex	Age*	Origin	Subspecies	Parameter η
4	Nicky	Male	29	Wild born	Schweinfurthii	Yes
4	Sinkie	Male	26	Wild born	Schweinfurthii	Yes
4	Bobby	Male	26	Wild born	Schweinfurthii	Yes
4	Kambo	Female	24	Wild born	Schweinfurthii	Yes
4	Kathy	Female	21	Wild born	Conflicting results	Yes
4	Miracle	Female	20	Captive born	Schweinfurthii	Yes
4	Val	Male	20	Wild born	Schweinfurthii	Yes
4	Commander	Male	20	Wild born	Schweinfurthii	Yes
4	Kit	Male	15	Captive born	Unknown	Yes
4	Jack	Male	12	Captive born	Unknown	No
4	Leila	Female	9	Wild born	Unknown	No
4	Ken	Male	8	Captive born	Unknown	Yes
4	Jewel	Male	6	Captive born	Unknown	No
4	Grace	Female	5	Wild born	Unknown	No

Table S4: Group 4 info. *Age at the end of the observation window (late 2019)

$\chi(t)$ fittings

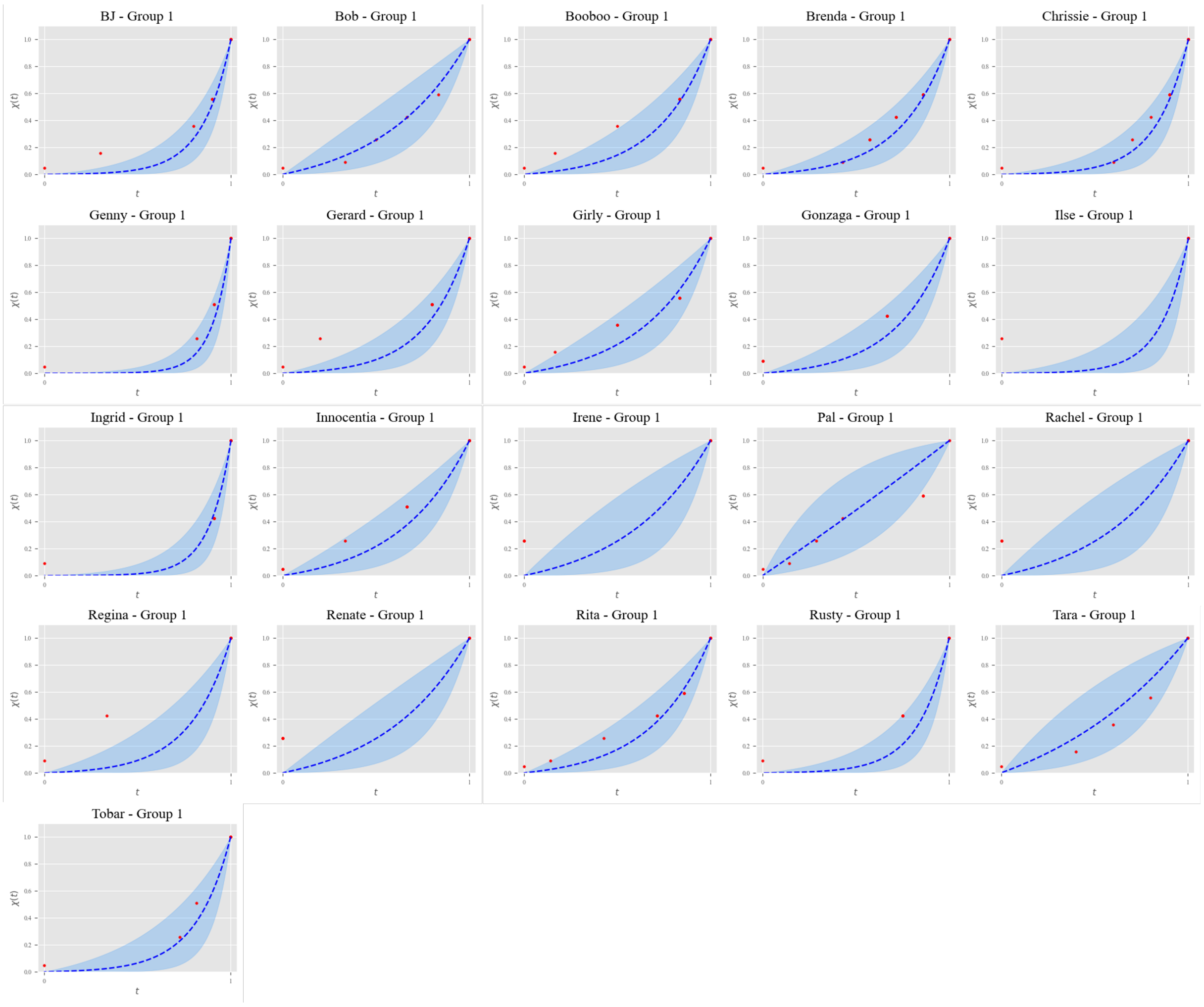

Figure S1: Group 1

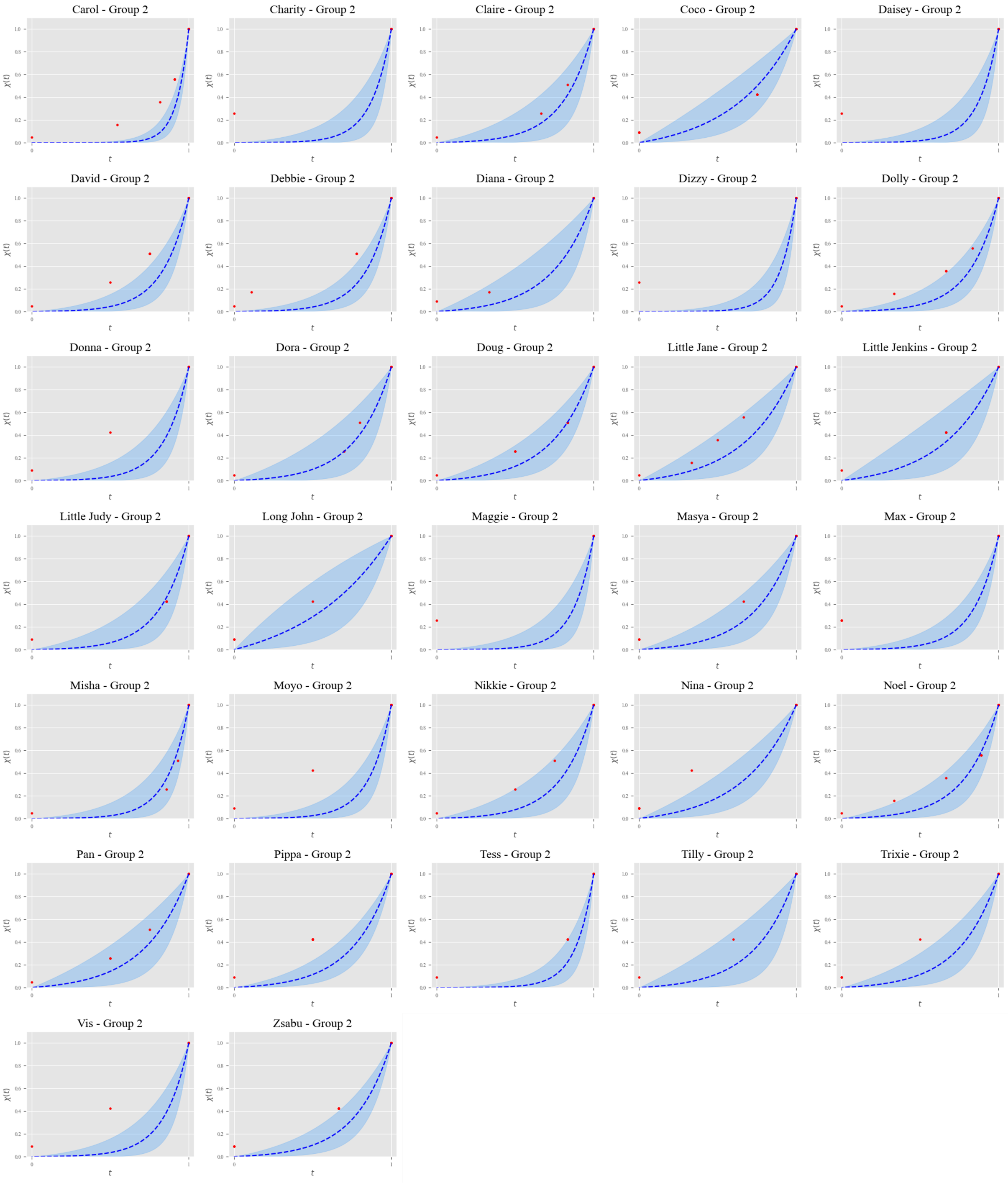

Figure S2: Group 2

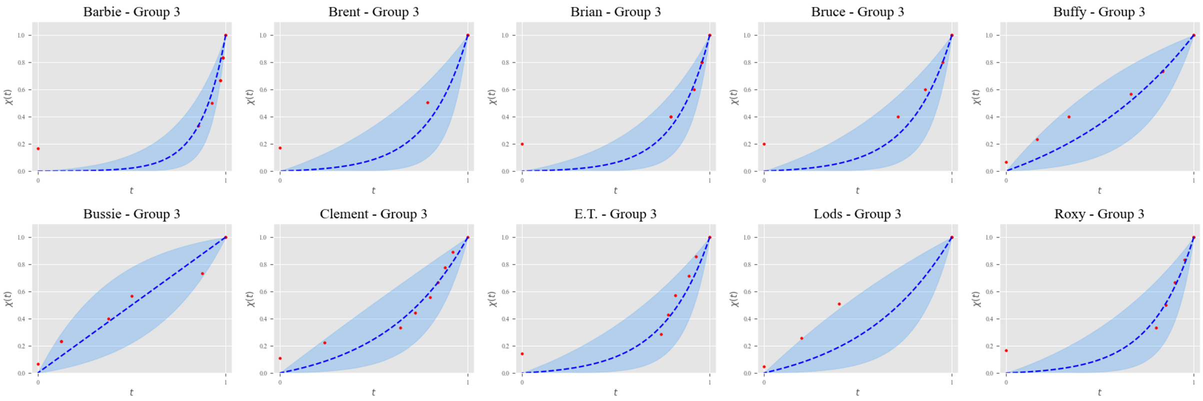

Figure S3: Group 3

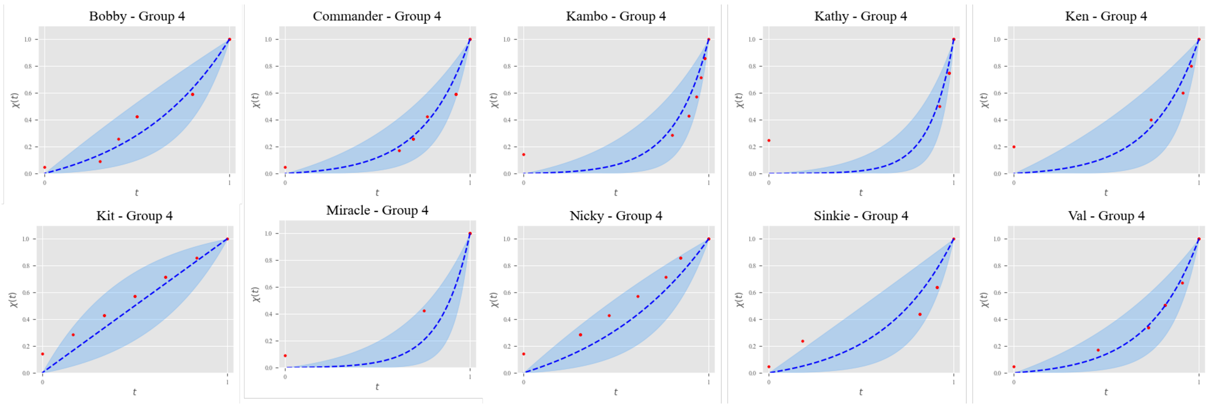

Figure S4: Group 4
